# Strategic Care of Ballistic Injuries: A Retrospective Observational Study at a Moroccan Military Hospital

**DOI:** 10.7759/cureus.85162

**Published:** 2025-05-31

**Authors:** S. Khalkane, Monsef Elabdi, Issam Serghini, Youssef Qamouss, Rachid Seddiki

**Affiliations:** 1 Cardiovascular Anesthesiology, Hopital Militaire d'instruction MedV, Rabat, MAR; 2 Trauma and Orthopedics, Hassan II Military Hospital, Laayoune, MAR; 3 Emergency Service, Avicenne Military Hospital, Marrakesh, MAR; 4 Anesthesia and Critical Care, Avicenne Military Hospital/Cadi Ayyad University, Marrakesh, MAR

**Keywords:** ballistic trauma, damage control resuscitation, damage control surgery, military medicine, trauma care protocols

## Abstract

Background

Ballistic trauma from high-velocity projectiles causes severe multisystem injury and rapid physiological decline. While well-characterized in combat and civilian violence, accidental firearm injuries during military training remain understudied. These incidents typically affect young, unprotected individuals in settings with limited prehospital care factors that may influence injury patterns and outcomes. This study aims to describe the clinical features, injury profiles, and management of accidental ballistic trauma in a military training context, with implications for prevention, triage, and critical care in resource-limited settings.

Methods

We conducted a six-year retrospective observational study (2016-2021) at Hassan II Military Hospital, Morocco, including 32 consecutive male patients admitted to the intensive care unit (ICU) for accidental high-velocity ballistic trauma sustained during military training. Injuries were caused by North Atlantic Treaty Organization (NATO)-standard live ammunition (5.56 × 45 mm or 7.62 × 51 mm). No exclusions were applied. Data collected included demographics, injury characteristics, prehospital care, hemodynamic parameters at admission, surgeries, and outcomes. ICU admission followed institutional protocols based on hemodynamic instability, high trauma burden, or need for invasive support. Outcomes were classified as favorable (ICU discharge without major complications) or unfavorable (mortality, organ failure, or reoperation). The study received ethics approval from the Institutional Committee of Hassan II Military Hospital.

Results

The mean age was 30.7 years; half of the patients were aged 31-40. Multiregional injuries were frequent: limb trauma (50%), abdominal injury (37.5%), and neurological impairment (Glasgow Coma Scale (GCS): 9-13 in 37.5%). On ICU admission, tachypnea (62.5%), tachycardia (50%), and hypotension (37.5%) were common. Most patients (87.5%) underwent surgery (60% orthopedic and 40% abdominal), with a mean delay of 13.2 ± 7.3 hours, mainly due to prehospital transfer and imaging delays. Intensive monitoring was required in 62.5%. Injury Severity Score (ISS) ranged from 12 to 32, indicating moderate-to-severe trauma in over one-third of patients. Favorable outcomes were observed in 62.5%. Unfavorable outcomes (37.5%), including mortality and reoperation, were significantly associated with older age, comorbidities, hypotension, mean arterial pressure (MAP) < 65 mmHg, and oliguria (p < 0.05).

Conclusion

Accidental ballistic trauma during military training is associated with significant morbidity and mortality, particularly when early hemodynamic instability is present. Findings support early triage, aggressive resuscitation, and application of damage control principles at the point of injury ("damage control at ground zero") as critical strategies. Although limited by its retrospective, single-center design and small sample, this study provides foundational evidence to guide trauma protocols and improve care delivery in resource-limited military environments.

## Introduction

Gunshot injuries are associated with severe consequences, including extensive tissue destruction, infection, and hemorrhage. Armed violence continues to pose a significant public health burden, particularly in developing regions where illicit firearm circulation and armed conflicts are prevalent. Recent global conflicts, such as the war in Ukraine since February 24, 2022, and recurrent violence in Gaza, have underscored the devastating impact of ballistic trauma on civilian populations and the urgent need for advancements in trauma care [1,2].

Severe ballistic injuries, including gunshot and blast wounds, frequently result in complex damage to soft tissues, bones, and vital organs, requiring rapid surgical intervention. Management challenges are particularly pronounced in conflict settings, where head and thoracic injuries are associated with high morbidity and mortality rates [2]. Expertise in treating ballistic trauma is essential to optimize outcomes, especially in resource-constrained environments.

Damage control surgery (DCS) has emerged as a cornerstone of early trauma management, prioritizing hemorrhage control, temporary stabilization of fractures, and early debridement. Treatment principles, drawn from prior conflicts in Iraq and Afghanistan, emphasize the importance of swift prehospital interventions and multidisciplinary collaboration [3]. Adjunctive therapies, such as the early administration of tranexamic acid (TXA), have been validated by large trials such as CRASH-2, demonstrating significant mortality reduction through prompt control of bleeding [4].

In this context, our study aimed to evaluate and improve the management of ballistic trauma at Hassan II Military Hospital over a six-year period. Through a retrospective analysis of 32 patients, we sought to describe injury patterns, prehospital interventions, surgical strategies (including delayed closure and endovascular techniques), and the integration of ultrasound-guided regional anesthesia to enhance perioperative care and minimize opioid requirements.

## Materials and methods

We conducted a retrospective study at Hassan II Military Hospital (Morocco) over a six-year period (2016-2021), including 32 consecutive male patients admitted to the intensive care unit (ICU) for accidental high-velocity ballistic trauma during military training. All cases involved live North Atlantic Treaty Organization (NATO)-standard ammunition (5.56 × 45 mm or 7.62 × 51 mm), with no exclusions based on injury severity or site. Data included demographics, injury profiles, prehospital care, hemodynamics, surgeries, and outcomes. ICU admission followed institutional criteria, such as instability, major trauma, or need for invasive support. Favorable outcomes were ICU discharge without major complications; unfavorable outcomes included death, organ failure, or reoperation. Ethical approval was granted by the Ethics Committee of Hassan II Military Hospital.

Study objective

The primary objective of this study was to systematically characterize the epidemiological profiles, anatomical injury patterns, and multidisciplinary management strategies of ballistic trauma sustained during military training exercises, specifically among patients admitted to the ICU. The secondary objective was to identify early prognostic indicators, such as age, hemodynamic instability, and injury severity, associated with unfavorable clinical outcomes.

Study design and setting

This single-center, retrospective observational study was conducted at Hassan II Military Hospital, a tertiary-level military referral center in Morocco, over a six-year period from January 1, 2016, to December 31, 2021. The study protocol was reviewed and approved by the Institutional Ethics Committee of Hassan II Military Hospital and conducted in full compliance with institutional standards and international research guidelines, including the revised Declaration of Helsinki.

Study population

This study included 32 consecutive male patients, aged 18-60 years, who sustained accidental ballistic injuries during official military training exercises and were subsequently admitted to the ICU. Inclusion criteria mandated documented ICU admission for trauma, complete clinical, imaging, and surgical records, and written consent for anonymized data use. Patients with non-trauma-related ICU admissions or incomplete medical documentation were excluded during initial screening. One misclassified non-ICU case was identified and excluded following a systematic review to ensure methodological integrity.

Data collection and operational definitions

Clinical data were retrospectively extracted from both electronic and paper-based medical records using a standardized abstraction form developed and pilot-tested by the clinical research team. All reviewers received structured training before data collection to ensure methodological consistency. Data abstraction was performed in parallel by two independent physician reviewers, with discrepancies resolved through consensus.

Collected variables included demographics, injury characteristics (mechanism, anatomical location, and entry and exit wounds), prehospital interventions, physiological parameters upon ICU admission (vital signs, hemodynamic, and respiratory status), radiologic findings (X-ray, CT, and ultrasound), intraoperative and medical interventions, and clinical outcomes (ICU length of stay, complications, and in-hospital mortality).

Operational definitions were uniformly applied to enhance reliability. Hemodynamic instability was defined as systolic blood pressure < 90 mmHg, mean arterial pressure (MAP) < 65 mmHg, or vasopressor requirement upon ICU admission. Oliguria was defined as urine output < 0.5 mL/kg/h.

Complications included clinically confirmed infections requiring antimicrobial therapy, unplanned reoperations, prolonged mechanical ventilation (>48 hours), or organ dysfunction (renal, hepatic, or respiratory) as per standard ICU definitions. Favorable outcome was defined as survival to hospital discharge without major complications or irreversible functional impairment.

Inclusion criteria were male patients aged 18-60 years, who were admitted to the ICU for ballistic trauma sustained during military training exercises, with complete clinical, radiological, and operative documentation, and documented consent for the use of anonymized data.

While full radiological imaging (pre- and postoperative) was preferred, some cases with complete perioperative clinical assessment were included despite partial imaging data or incomplete short- and medium-term follow-up to retain cases with valid acute-phase therapeutic and prognostic value.

Exclusion criteria encompassed ICU admissions for non-traumatic conditions and cases with incomplete records, specifically those lacking critical preoperative or postoperative imaging or essential physiological data, which were excluded based on failure to meet eligibility criteria during the systematic review.

Cases not meeting predefined inclusion criteria were excluded during the initial screening phase. Only patients with complete documentation of core clinical variables were retained for final analysis, and no data imputation was employed to preserve analytical integrity. After a systematic review of ICU trauma logs, 32 consecutive male patients with confirmed ballistic injuries sustained during military training and admitted to the ICU were included. One case initially misclassified as ICU-admitted was excluded following a post-hoc protocol audit to ensure methodological rigor and dataset consistency.

Trauma severity was retrospectively assessed using standardized classification systems, including the Injury Severity Score (ISS), the Mangled Extremity Severity Score (MESS), the Revised Trauma Score (RTS), and the Glasgow Coma Scale (GCS), where data permitted. Although formal interrater reliability testing was not conducted, all scores were independently assigned by two trauma-trained clinicians, with discrepancies resolved through consensus, thereby enhancing internal consistency within the constraints of a retrospective design. These severity indices were applied to support injury stratification and facilitate external benchmarking with comparable trauma datasets.

Statistical analysis

Statistical analyses were conducted using IBM SPSS Statistics, version 25.0 (IBM Corp., Armonk, NY). The normality of continuous variables was assessed using the Shapiro-Wilk test. Normally distributed data were presented as means ± standard deviations (SD), while non-normally distributed data were reported as medians with interquartile ranges (IQR). Categorical variables were summarized as absolute counts and percentages. Comparative analyses between patients with favorable and unfavorable outcomes were performed using the student’s t-test or Mann-Whitney U test for continuous variables, and the Chi-square or Fisher’s exact test for categorical variables. Binary associations were expressed as odds ratios (ORs) with corresponding 95% confidence intervals (CIs). All statistical tests were two-tailed, with a significance threshold of p < 0.05. Due to the limited sample size (n = 32), multivariate logistic regression was not performed to avoid model overfitting and ensure analytical reliability; this limitation is explicitly addressed in the discussion. To contextualize findings, a targeted narrative literature review was conducted using PubMed, Google Scholar, and the Cochrane Library, encompassing relevant English- and French-language publications available up to April 2022. Search terms included “ballistic trauma,” “military injuries,” “damage control surgery,” “gunshot wounds,” and “trauma during military training.” Studies were selected based on their methodological rigor, clinical relevance, and applicability to the military or resource-limited trauma setting. Although no formal meta-analysis was performed, the literature review offered critical insights into contemporary best practices in damage control resuscitation, operative strategies, and outcome determinants within military trauma systems, thereby supporting the interpretation and external positioning of the present study's findings.

Bias mitigation and methodological rigor

To mitigate the inherent limitations of retrospective research, several methodological safeguards were implemented. Selection bias was reduced by including all consecutive ICU-admitted ballistic trauma patients who met predefined inclusion criteria during the study period. Information bias was addressed through the use of a standardized, prevalidated data abstraction form and independent double-review of each case, with discrepancies resolved by consensus. Data consistency was further enhanced by structured reviewer training. Nonetheless, the retrospective design carries unavoidable risks of underreporting and variable completeness, particularly concerning prehospital interventions and early imaging. Additionally, the absence of long-term follow-up and rehabilitation metrics was explicitly acknowledged as a limitation in the “Discussion” section.

Ethical oversight

This study received formal approval from the Institutional Ethics Committee of Hassan II Military Hospital, Morocco (IRB No.: M-MAROC-26-78-REG4/Ethics Approval No.: M-123-MAR; Approval Date: February 12, 2024). All research procedures were conducted in accordance with the Moroccan National Regulatory Requirements, Institutional Ethical Standards, and the principles outlined in the revised Declaration of Helsinki (2013).

Selected case descriptions

**Figure 1:**
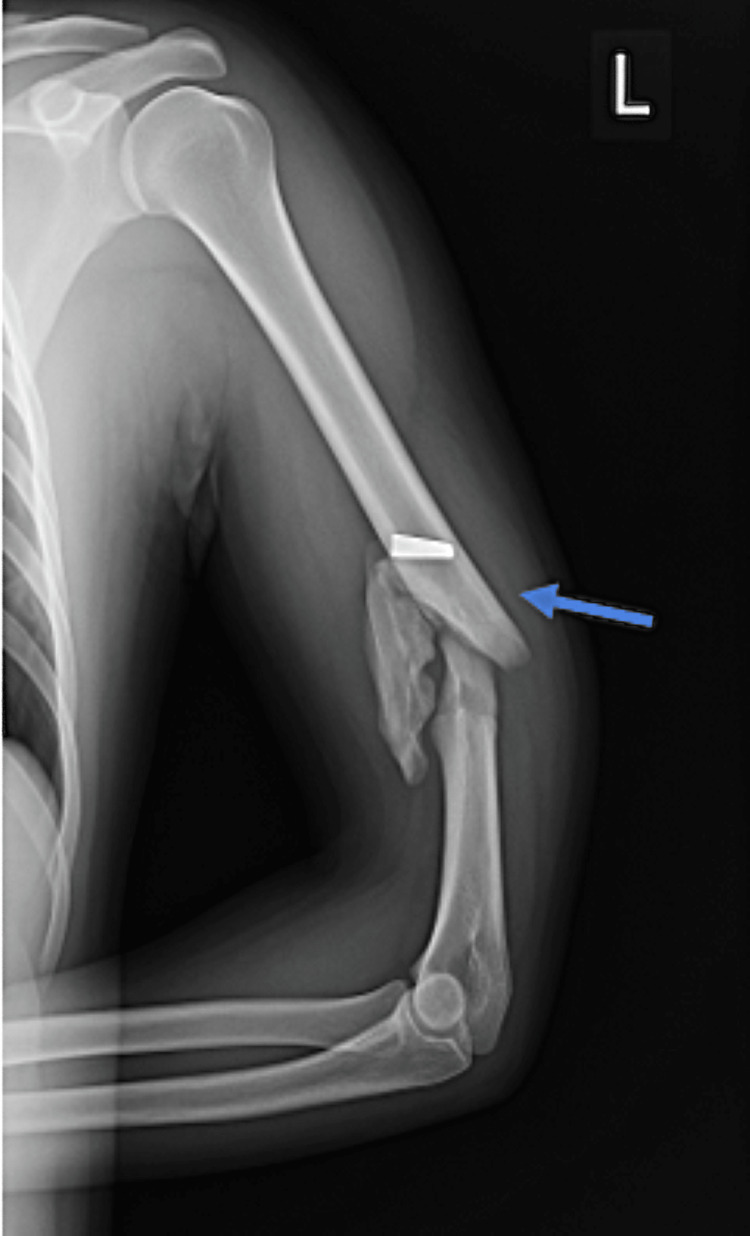
Radiograph of the upper limb demonstrating a comminuted mid-diaphyseal fracture of the humerus (blue arrow) with a retained bullet fragment lodged within the soft tissues of the arm

To illustrate the clinical heterogeneity and complexity of ballistic trauma sustained during military training, we present nine representative ICU-admitted cases, selected based on hemodynamic instability or multisystem injuries and complete core documentation with available imaging. In Case 1, a 19-year-old male sustained a left upper arm gunshot wound during a live-fire exercise, presenting with active bleeding and impaired mobility. Imaging revealed a Gustilo-Anderson Type IIIA open distal humeral fracture with a retained extra-articular bullet (Figure 1). Though initially stable, he developed transient hypotension and tachycardia, prompting ICU admission for monitoring and early intervention. He received tetanus prophylaxis and IV antibiotics, and underwent urgent surgical debridement with internal fixation using a distal humerus locking plate; the bullet was left in situ due to its stable location. Postoperative recovery was uneventful, and the patient was discharged on a structured rehabilitation plan with favorable radiographic and functional outcomes at six weeks.

**Figure 2:**
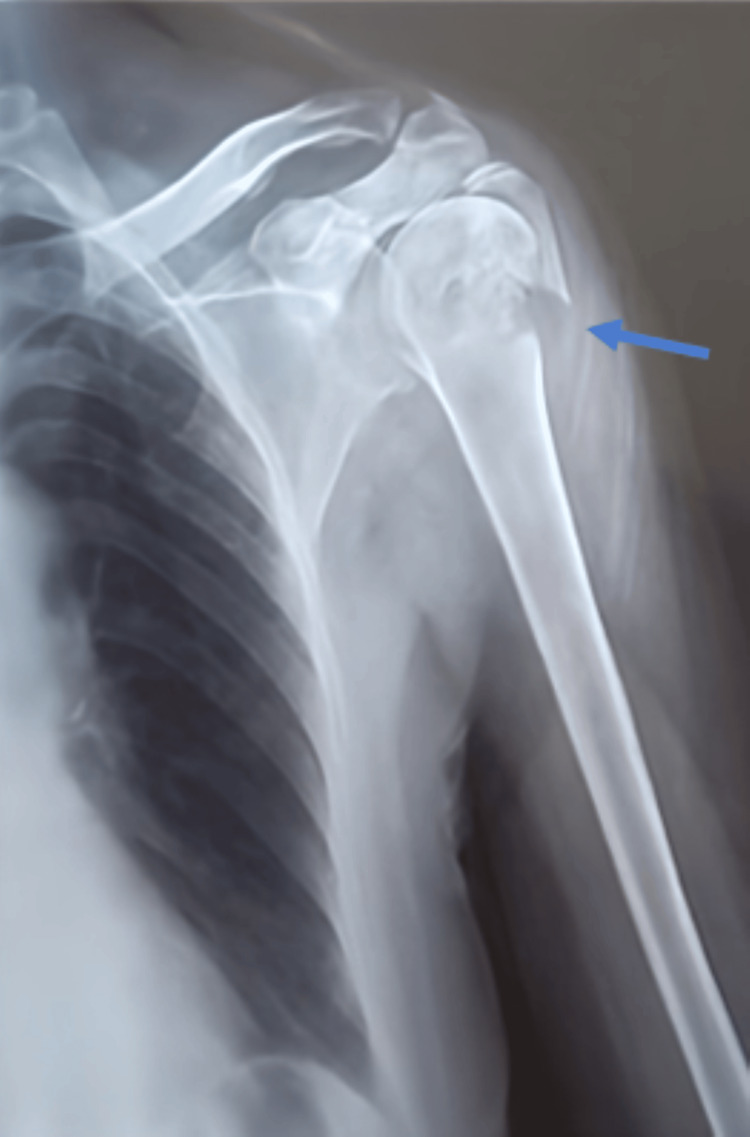
Standard frontal X-ray of the left shoulder showing a complex comminuted fracture-dislocation at the basicervical and humeral head regions with posterior lateral avulsion of a fracture fragment (blue arrow), with infiltration of the adjacent soft tissues

Case 2 involved a 22-year-old male with a high-velocity gunshot wound to the left shoulder, resulting in a comminuted basicervical humeral fracture with associated glenohumeral dislocation and posterolateral avulsion (Figure 2). Despite initial hemodynamic stability, ICU admission was warranted for close neurologic and vascular monitoring, effective pain control, and anticipation of potential complications such as compartment syndrome. The radiographic assessment confirmed extensive articular and periarticular disruption. The patient underwent open reduction, internal fixation, and bullet tract debridement. The postoperative course was uncomplicated, and structured rehabilitation facilitated satisfactory recovery, with restored joint mobility and strength noted at follow-up.

**Figure 3:**
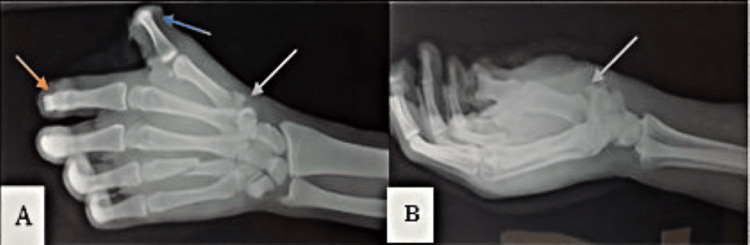
Standard radiographs of the hand. (A) Anteroposterior view and (B) lateral view, demonstrating the absence of the distal phalanges of the thumb (blue arrow) and second digit (orange arrow), with associated soft tissue loss. A midshaft fracture of the fourth metacarpal and a fracture at the base of the first metacarpal (gray arrow) are also noted.

Case 3 involved a 27-year-old male who sustained a direct gunshot wound to the left hand during live-fire training. On arrival, he presented with extensive soft tissue loss, traumatic amputations of the distal phalanges of the thumb and index finger, and visible bone fragments. Imaging showed destruction of the distal rays, a midshaft fracture of the fourth metacarpal, and a fracture at the base of the first metacarpal (Figure 3). After ICU admission for pain control and wound management, he received tetanus prophylaxis and broad-spectrum IV antibiotics. Urgent surgical intervention included thorough debridement, internal fixation of the fourth metacarpal, and stump revision. Conservative management was chosen for the base of the first metacarpal due to the surrounding soft tissue compromise. The patient was later referred to a specialized hand surgery unit for staged reconstructive procedures. This case underscores the functional impact of high-energy ballistic hand trauma and the critical need for multidisciplinary care and long-term rehabilitation planning.

**Figure 4:**
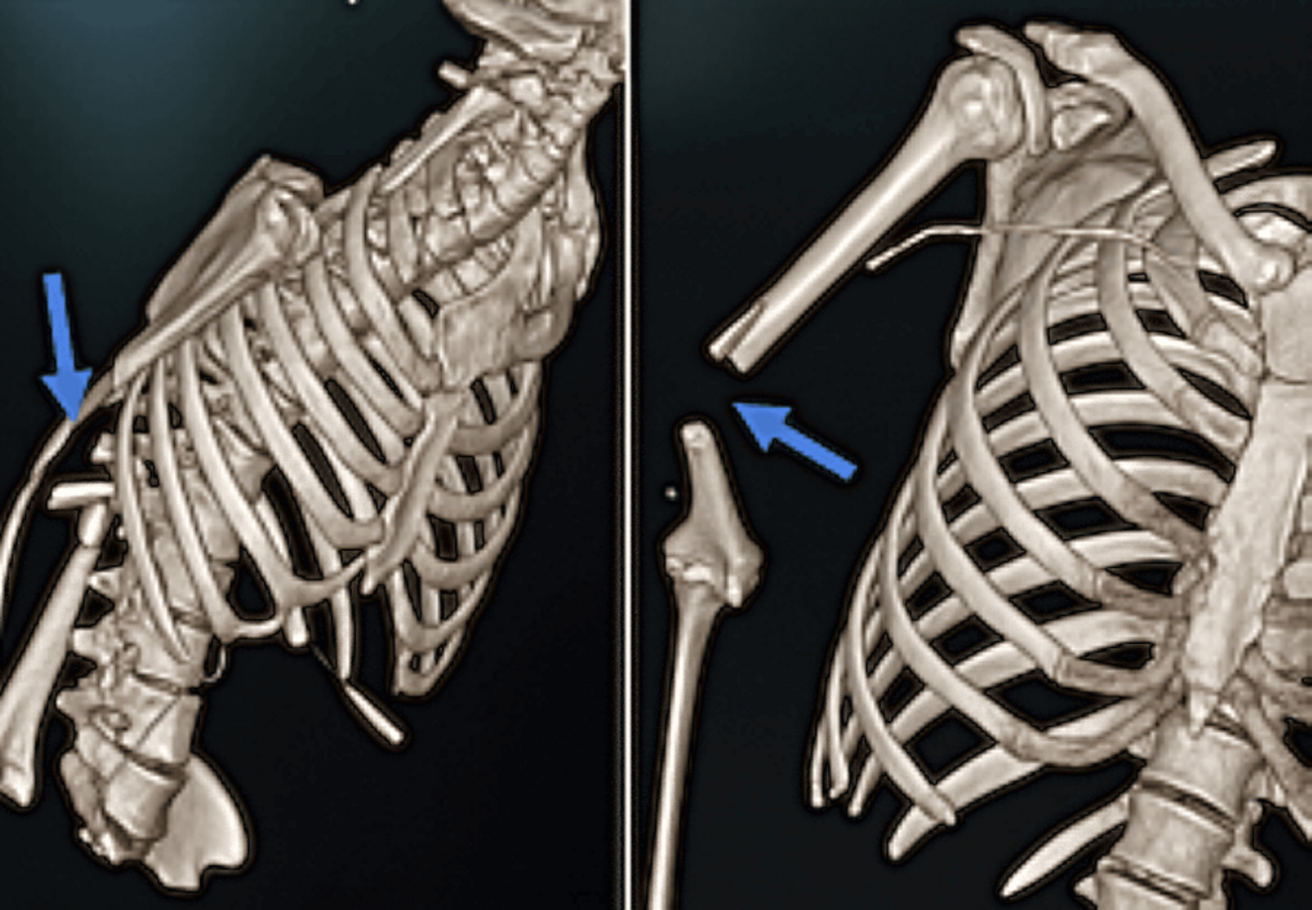
Three-dimensional reconstruction from a CT scan of the right upper limb showing an open diaphyseal fracture of the distal third of the humerus, characterized by a transverse fracture line and marked anterolateral displacement of the fracture fragments (blue arrows).

In Case 4, a 35-year-old artillery trainee sustained a high-velocity gunshot injury to the right upper limb, presenting with gross deformity, active bleeding, and restricted mobility, but preserved distal perfusion and neurological function. Three-dimensional CT imaging confirmed a transverse distal humeral diaphyseal fracture with anterolateral displacement and cortical fragmentation, consistent with a Gustilo-Anderson Type IIIA open fracture (Figure 4). He was admitted to the ICU for monitoring and underwent urgent surgical debridement followed by open reduction and internal fixation with a locking plate. Perioperative management included tetanus prophylaxis and broad-spectrum antibiotics. Recovery was uncomplicated, and early physiotherapy contributed to restored limb alignment and mobility, as confirmed by follow-up imaging at six weeks. This case highlights the value of 3D imaging in surgical planning and reinforces the importance of timely orthopedic intervention in high-energy open fractures.

**Figure 5:**
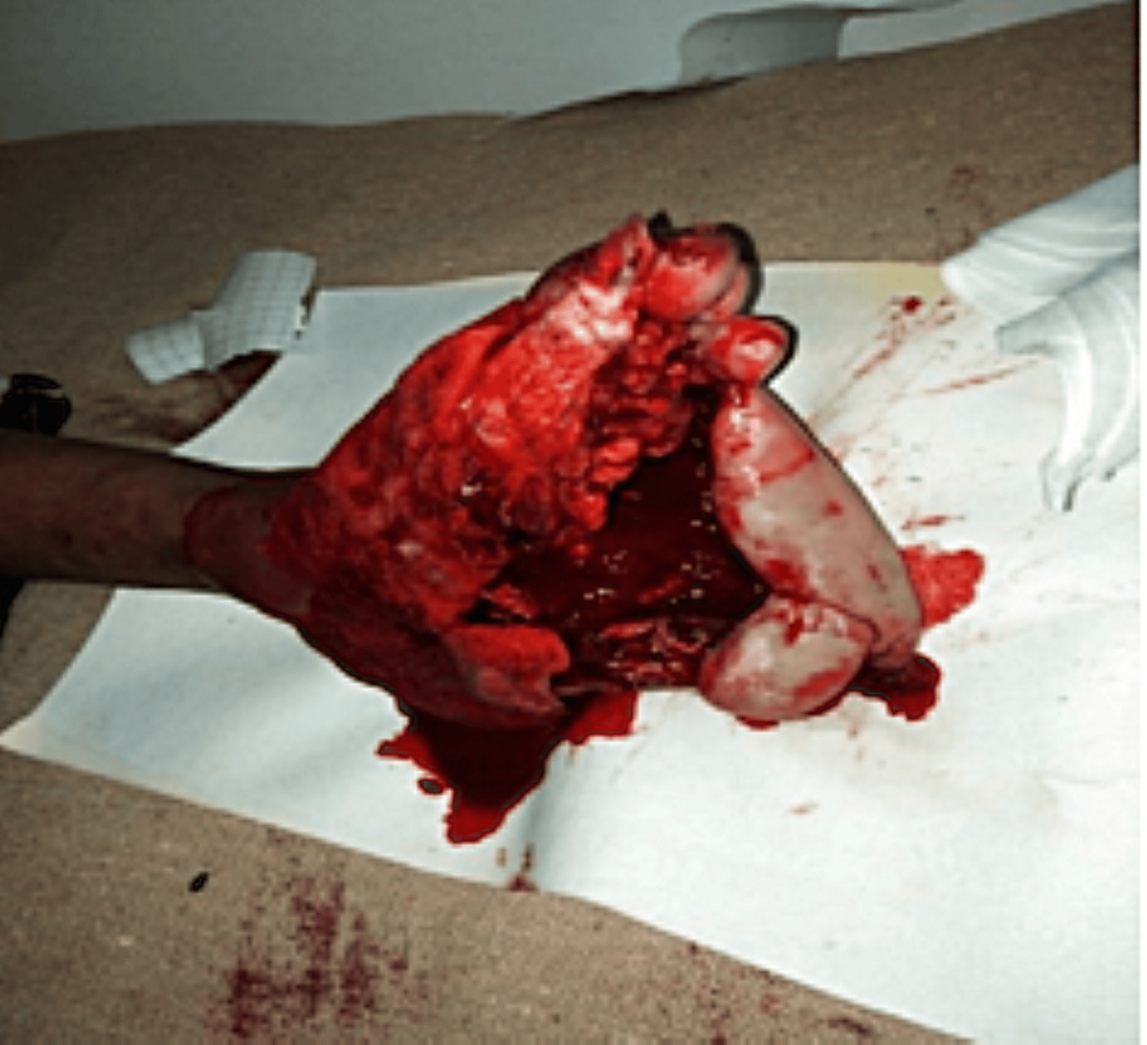
Photograph of the hand demonstrating extensive osseous and soft tissue damage following a blast injury sustained while handling an explosive device

**Figure 6:**
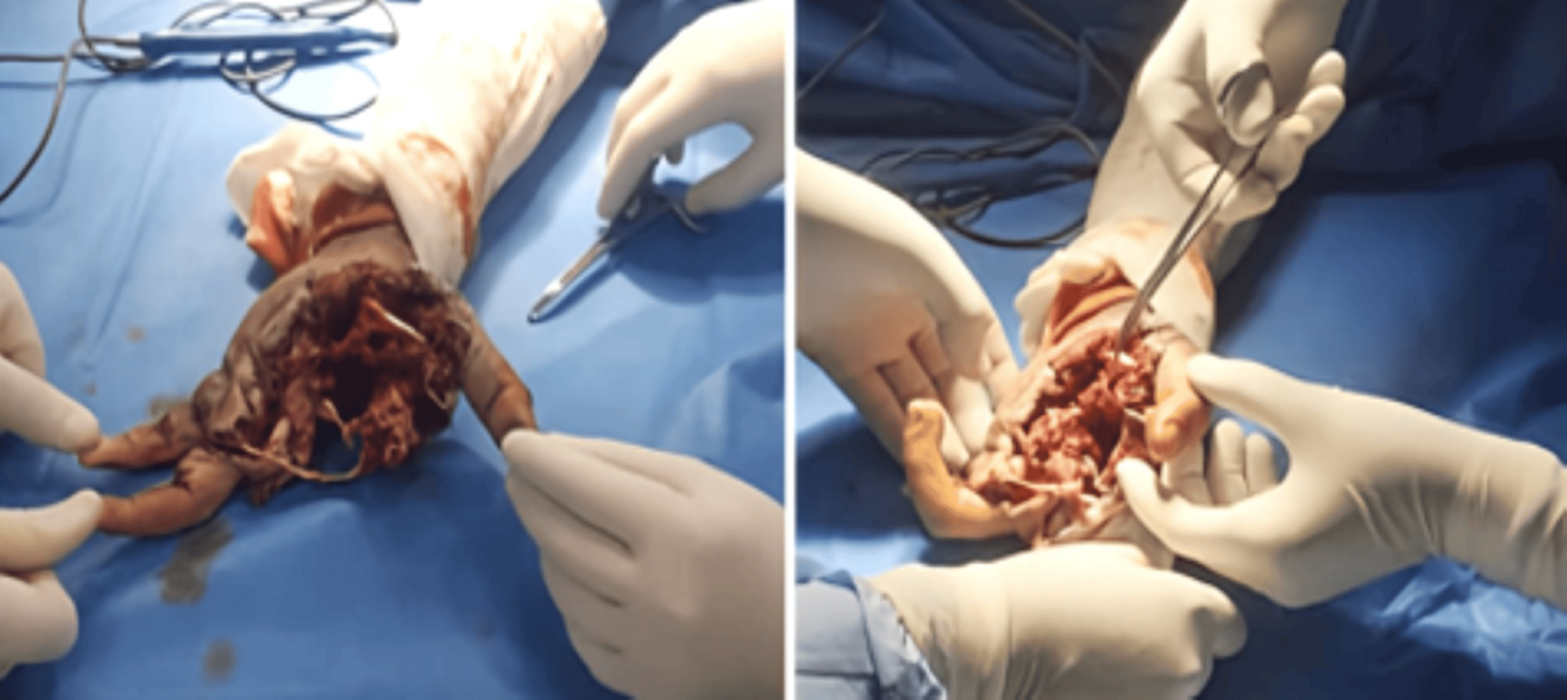
Intraoperative photograph showing surgical management of a blast-related hand injury, with a lesion pattern predominantly affecting the first web space

Case 5 featured a 31-year-old right-handed military trainee who sustained a blast injury to the right hand following an accidental detonation of an explosive device. He remained conscious and hemodynamically stable but presented with extensive tissue destruction, including open fractures, gross contamination, and avulsion injury of the first web space. Intraoperative findings revealed fragmentation of the first and second metacarpals, tearing of the adductor pollicis, and a vascular lesion requiring ligation (Figures 5, 6). Emergency surgical management consisted of debridement, external fixation, and delayed closure with regional flap coverage. Tetanus prophylaxis and broad-spectrum antibiotics were administered. Due to retrospective limitations, no radiographs were available, and case analysis was based on detailed clinical and operative records. The patient was referred to a reconstructive hand surgery center and underwent staged rehabilitation. This case exemplifies the devastating consequences of high-energy blast trauma to the hand and the importance of early multidisciplinary planning to optimize salvage and function.

**Figure 7:**
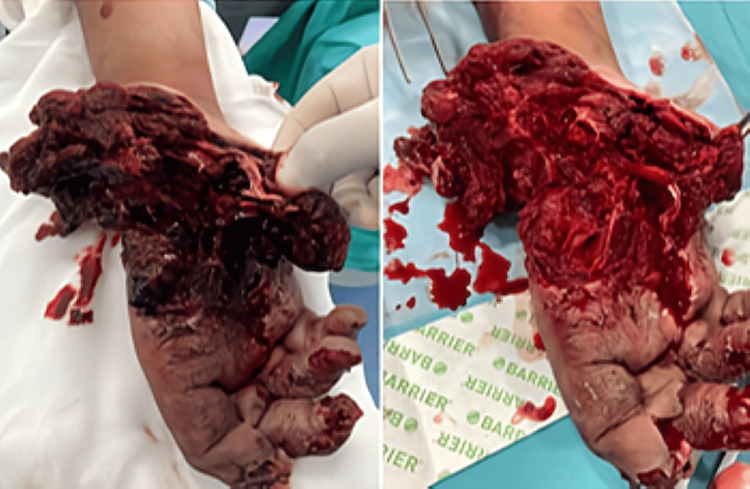
Photograph of the hand showing traumatic soft tissue loss on the ventral aspect with extensive injury to the first and second rays and destruction of the hypothenar region

Case 6 involved a 19-year-old male infantry artillery trainee who sustained a severe ballistic injury to the right hand following accidental firearm discharge. The trauma resulted in extensive soft tissue loss involving the first and second rays, destruction of the hypothenar eminence and palmar structures (Figure 7), and dorsal tissue loss over the metacarpophalangeal joints and first web space.

**Figure 8:**
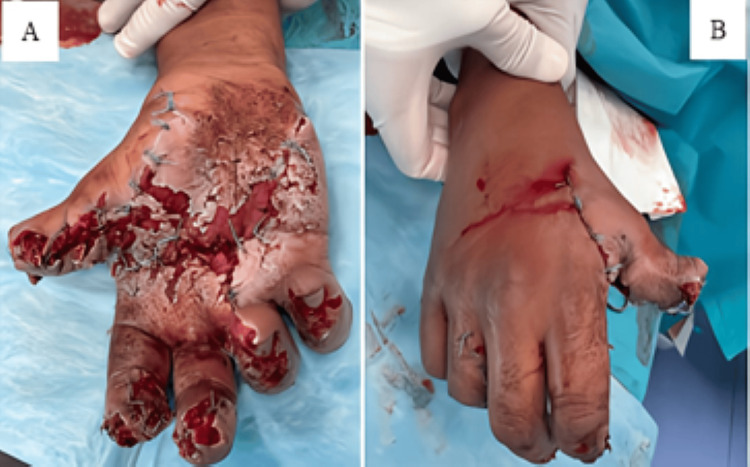
(A) Ventral view of the hand demonstrating a wound with loosely approximated sutures along the lesion margins. (B) Dorsal view showing loose sutures of the soft tissue over the metacarpophalangeal joints and within the first web space between the thumb and index finger.

The wound had been loosely approximated with sutures at the field station (Figure 8, Panel A). Due to the extent of anatomical disruption and absence of immediate imaging, urgent surgical debridement and wound revision were performed, followed by temporary closure and a plan for delayed reconstructive intervention (Figure 8, Panels A and B). The patient received IV antibiotics and tetanus prophylaxis and was subsequently referred to a tertiary hand surgery center. Despite an anatomically satisfactory result, functional recovery was limited due to median nerve injury, resulting in persistent flexion deficits and hypoesthesia in the radial digits. Two microsurgical interventions were performed with partial restoration of hand function. Formal rehabilitation outcomes were unavailable due to external follow-up. This case highlights the severity of ballistic hand trauma in young military personnel and the essential role of early multidisciplinary management, reconstructive planning, and long-term rehabilitation to optimize functional outcomes.

**Figure 9:**
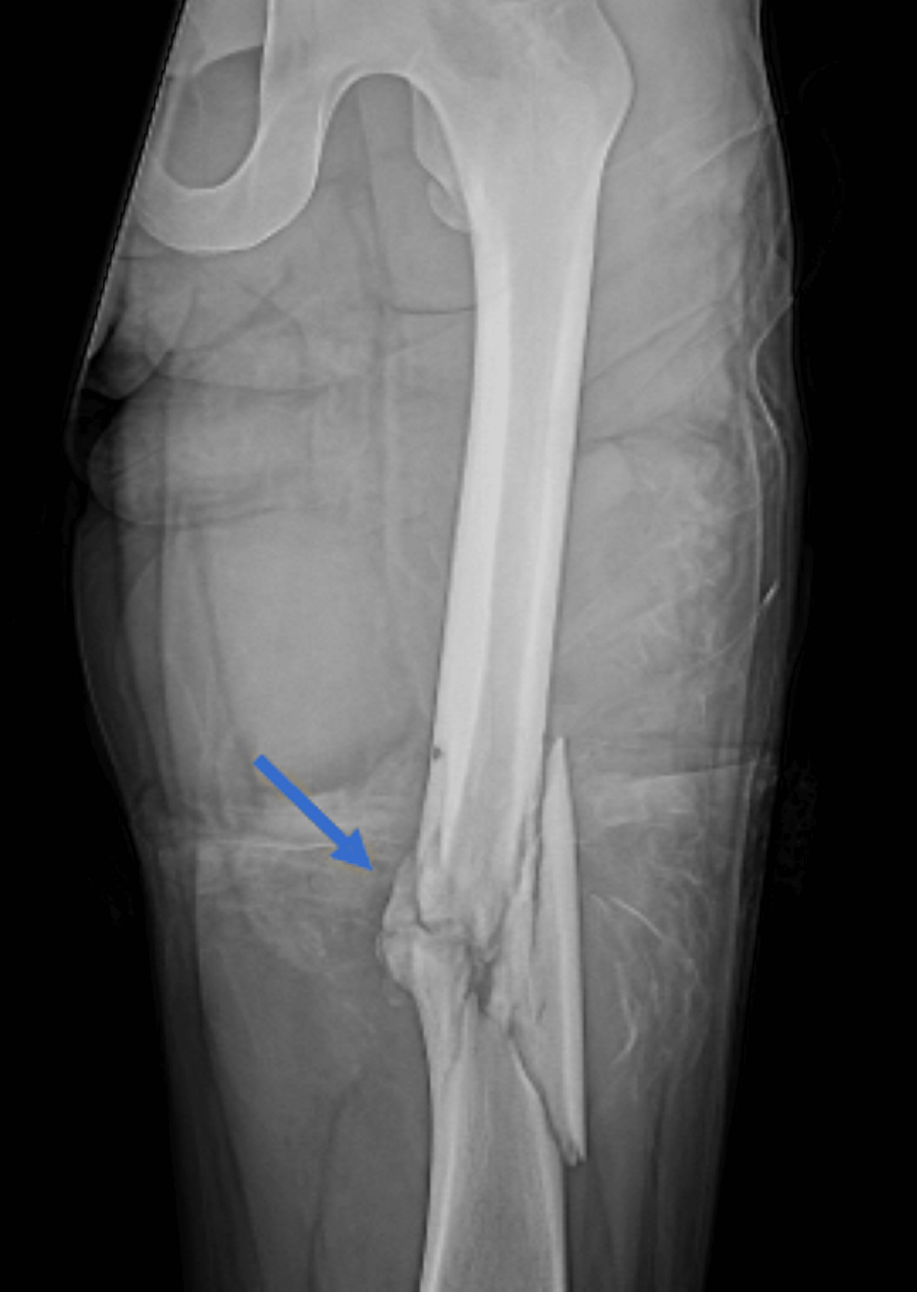
Frontal radiograph of the femur demonstrates a comminuted diaphyseal fracture involving the distal third of the femoral shaft (blue arrow)

Case 7 featured a 29-year-old with a high-energy ballistic femoral shaft fracture (Figure 9), successfully managed with debridement and intramedullary fixation.

**Figure 10:**
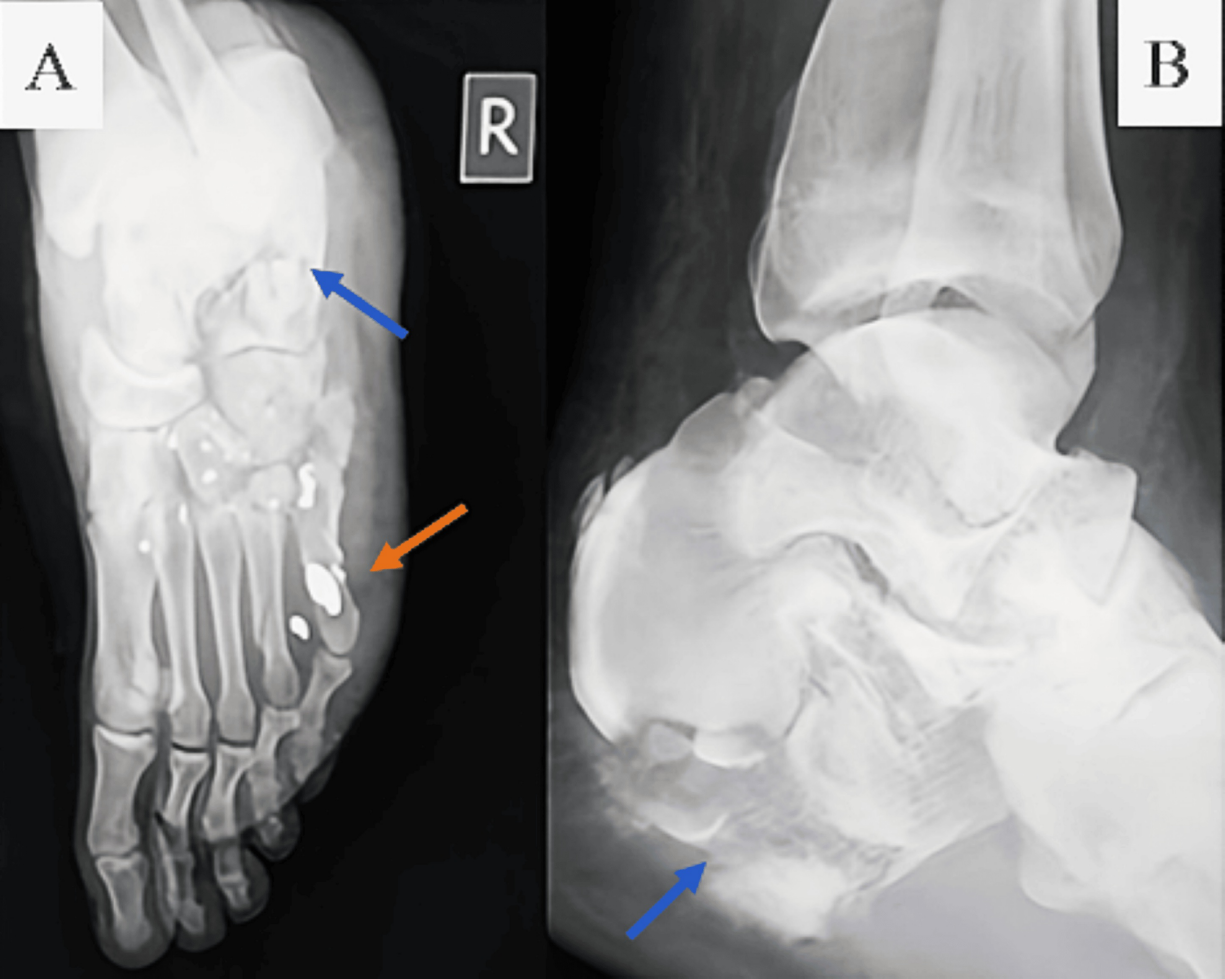
Standard radiographs of the right foot and ankle demonstrating a closed blast injury with comminuted calcaneal fracture. (A) The anteroposterior view shows extensive calcaneal fragmentation (blue arrow), with multiple radiodense foreign bodies consistent with metallic fragments from a blasting cap (orange arrow). (B) The lateral view confirms the comminuted nature of the calcaneal fracture.

Case 8 described a 21-year-old who sustained a calcaneal blast injury with embedded metallic fragments and systemic signs of shock (Figure 10), necessitating debridement, external fixation, and ICU monitoring.

**Figure 11:**
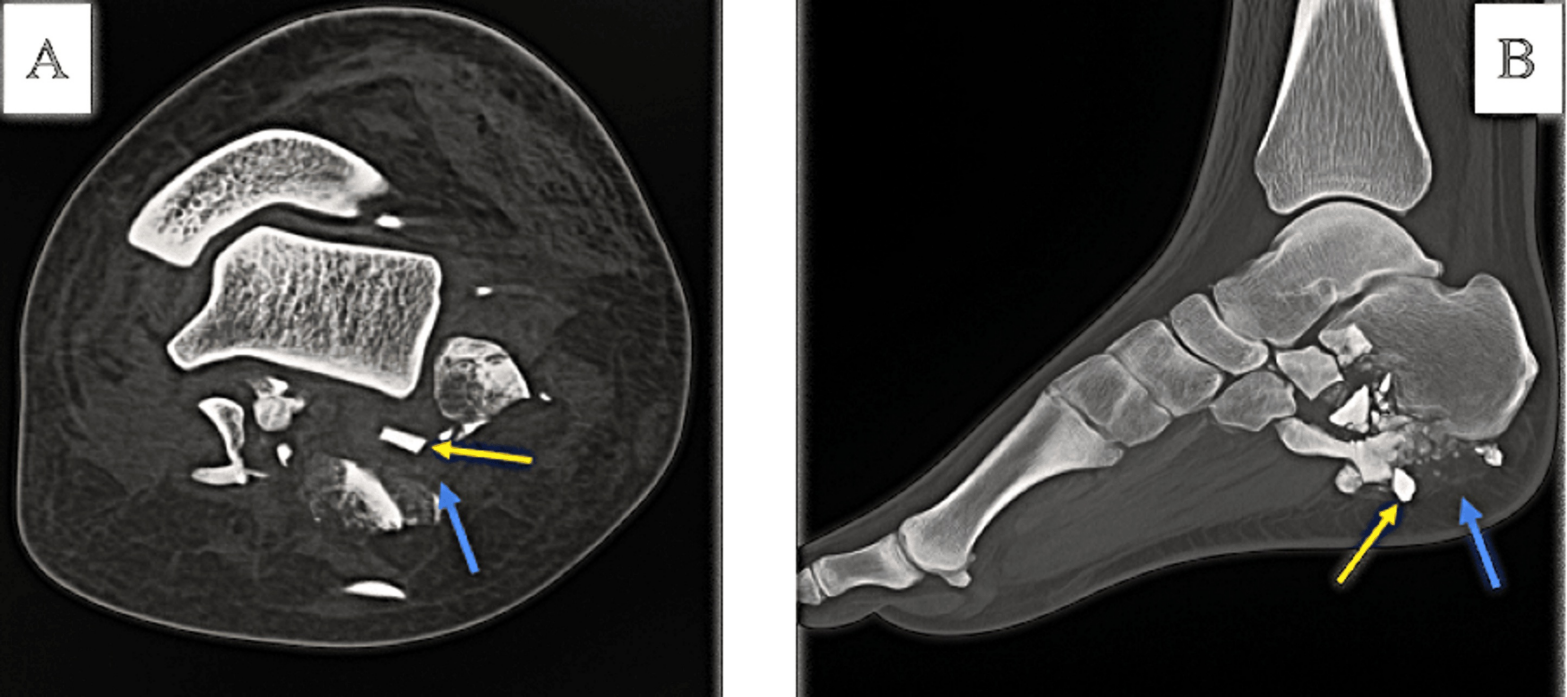
CT scan of the foot transversal view (A) and lateral view (B) with 3D reconstruction showing bone fragmentation of the calcaneus, reduced to small fragments with the destruction of the various intertarsal joint surfaces (talocalcaneal, calcaneo-navicular, and calcaneo-cuboid) (blue arrow) associated to small metallic foreign bodies within the bone fragments (blasting cap) (yellow arrow).

Finally, Case 9 involved a 33-year-old with a severe hindfoot blast injury, including joint destruction and compartment syndrome signs (Figure 11); he was managed with advanced imaging, debridement, temporary fixation, and referred for staged reconstruction. Together, these cases highlight the diversity of injury mechanisms, critical care requirements, and the importance of early surgical intervention and multidisciplinary management in military ballistic trauma.

## Results

Demographics and clinical characteristics

This retrospective cohort included 32 consecutive male patients, aged 18 to 42 years (mean ± SD: 30.7 ± 5.4 years), all admitted to the ICU following accidental ballistic trauma sustained during military training. The 31-40-year age group was most represented (50%, n = 16). A majority (75%, n = 24) had no chronic medical history; 25% (n = 8) were chronic smokers, and 15.6% (n = 5) had documented comorbidities, primarily hypertension and prior abdominal surgery. One case lacking documented ICU monitoring was excluded during final data validation to maintain methodological consistency. Notably, patients with unfavorable outcomes, defined as death or major complications, were significantly older than those with favorable outcomes (33.1 ± 6.2 vs. 29.5 ± 4.8 years; p < 0.05). Full demographic characteristics are provided in Table 1. The average delay from injury to hospital admission was 20.95 ± 10 hours (range: 1-65 hours).

**Table 1:** Baseline clinical characteristics of the study population

Parameters	Number (Frequency)
Age (Mean ± SD)	30.7 ± 5.4 years
Most affected age group	31–40 years (50%)
No prior medical history	20 (62.5%)
Hypertension	4 (12.5%)
Appendectomy history	1 (3.1%)
Chronic smoking	8 (25%)
Hemodynamic and respiratory stability	20 (62.5%)

Injury profiles and anatomic distribution

Limb trauma was the most prevalent injury type, observed in 50% of cases (n = 16), with 75% (n = 24) involving fractures equally distributed between the upper and lower extremities. Two patients sustained pelvic injuries classified as TILE A2 right iliac wing fractures. Abdominal trauma was documented in 37.5% of patients (n = 12), with presentations including abdominal distension, gastrointestinal bleeding, and evisceration. Neurological impairment, defined by a GCS score of 9-13, occurred in 37.5% of patients (n = 12), while spinal trauma, specifically D12 vertebral fractures with posterior displacement, was present in 12.5% of patients (n = 4). Detailed anatomical distributions are summarized in Tables 2, 3).

**Table 2:** Injury distribution and anatomical characteristics GCS: Glascow Coma Scale.

Characteristics	Number (Frequency)
Limb injuries	16 (50%)
Fractures among limb injuries	24 (75%)
Pelvic injuries (TILE A2)	2 (6.25%)
Abdominal distension	12 (37.5%)
Gastrointestinal bleeding	12 (37.5%)
Abdominal wounds	12 (37.5%)
Evisceration	4 (12.5%)
Neurological impairment (GCS: 9–13)	12 (37.5%)
Spinal trauma (D12 fractures)	4 (12.5%)
Functional limitation/pain	18 (56.5%)

**Table 3:** Anatomical distribution of entry and exit wounds in ballistic trauma patients

Case Number	Entry Wound Site	Exit Wound Site
1	Right iliac fossa	Pubis
2	Left iliac fossa	Left lumbar fossa
3	Left flank	Right iliac crest
4	Antero-external aspect of the left arm	Posterior aspect of the left arm
5	Antero-external aspect of the right shoulder	Postero-external aspect of the right shoulder
6	1st and 2nd rays + hypothenar region	Postero-external aspect of the hypothenar
7	Mid right arm, through the distal humerus	Laceration of the right elbow
8	Proximal palm of the right arm	Dorsal aspect of the hand
9	Multiple shrapnel wounds to the postero-internal right thigh	Absent
10	Anterior intercarpal face	Dorsal aspect of the hand
11	Lower third of the left arm with fracture	Postero-lateral aspect of the forearm
12	Anterior and lateral aspects of the left foot	Posterior aspect of the left foot
13	Outer aspect of the left calf	Posterior aspect of the left leg
14	Anterior aspect of the left leg	Posterior aspect of the left leg
15	Distal phalanx of the right second toe	Absent
16	Traumatic amputation of toes (3rd, 4th, and 5th)	Absent
17	Closed blunt injury to the chest	Absent
18	Traumatic leg amputation due to mine	Absent
19	Anterior arch of ribs 7 and 8	Absent
20	Lower level of fifth intercostal space	Posterior region
21	Right lower chest (seventh intercostal)	Absent
22	Abrupt wound to anterolateral region	Absent
23	Left flank	Left retrocostal area
24	Left iliac fossa	Posterior region
25	Left thigh + sacrum	Left retrocostal, ribs 6 and 7
26	Wound in the epigastric region	Absent
27	Right anterior subiliac	Right retrocostal region
28	Right flank	Right lumbar fossa
29	Left flank	Mid-dorsal level, T5–T6
30	Left subscapular region	Absent
31	Left anterolateral thorax	Absent
32	Left flank	Left lumbar fossa

Initial physiological status

Upon ICU admission, tachypnea and tachycardia were observed in 62.5% (n = 20) and 50% (n = 16) of patients, respectively. Hypotension, defined as systolic blood pressure < 90 mmHg or diastolic < 60 mmHg, was present in 37.5% of patients (n = 12), while both MAP < 65 mmHg and oliguria were documented in 25% (n = 8) of cases. Despite these abnormalities, oxygen saturation remained above 90% in 75% of the cohort (Table 4).

**Table 4:** ICU admission: physiological parameters SBP: Systolic blood pressure; DBP: Diastolic blood pressure; MAP: Mean arterial pressure.

Physiological Parameters	Percentage (%)
Tachypnea	62.5
Oxygen saturation > 90%	75.0
Hypotension (SBP < 90 or DBP < 60)	37.5
MAP < 65 mmHg	25.0
Tachycardia (>100 bpm)	50.0
Oliguria (<0.5 mL/kg/h)	25.0

Radiological and intraoperative findings

**Figure 12:**
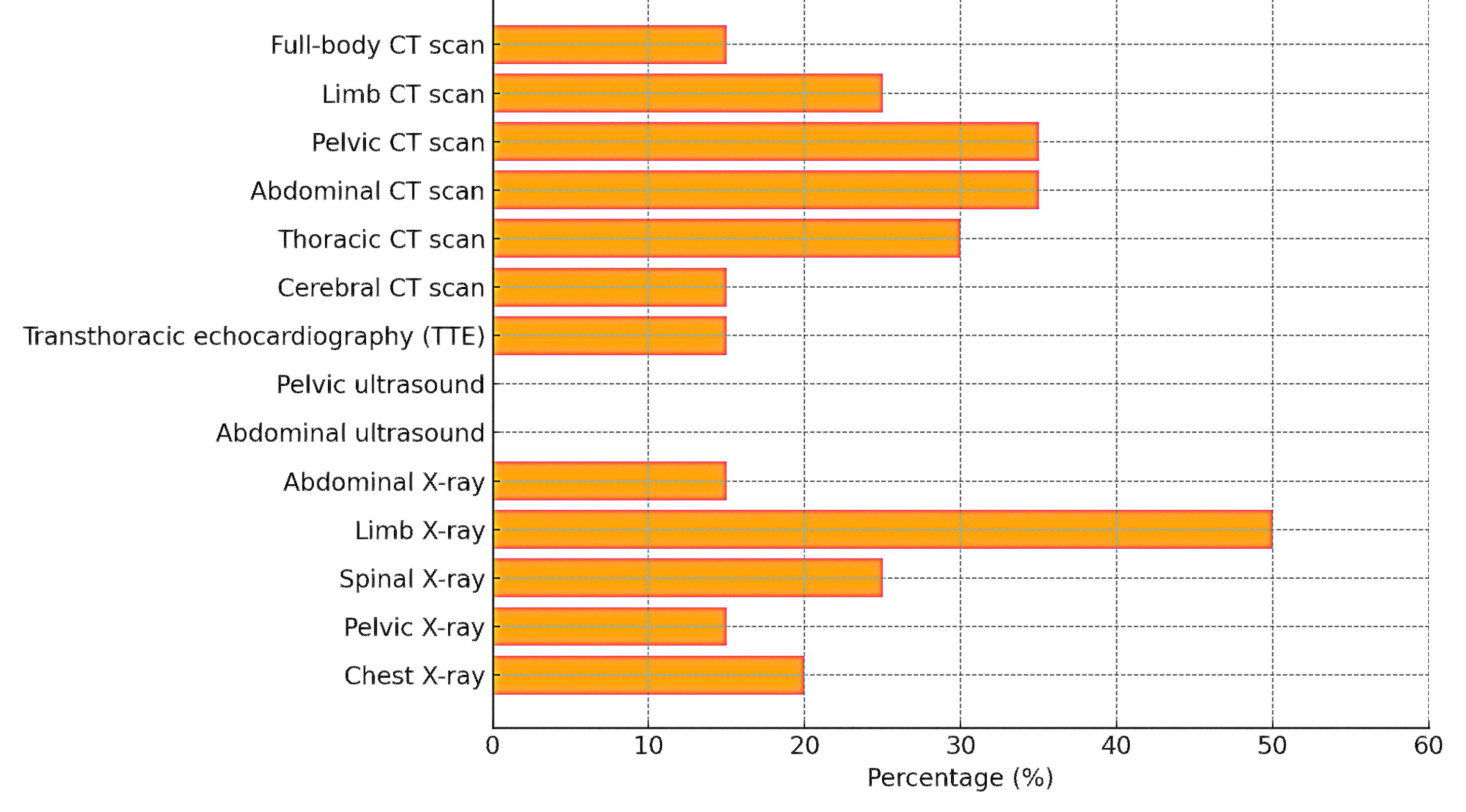
Distribution of radiological examinations performed on trauma patients

All patients underwent diagnostic imaging, including standard X-rays, transthoracic echocardiography, abdominal and pelvic ultrasound, and CT scans (Figure 12).

Therapeutic interventions

**Figure 13:**
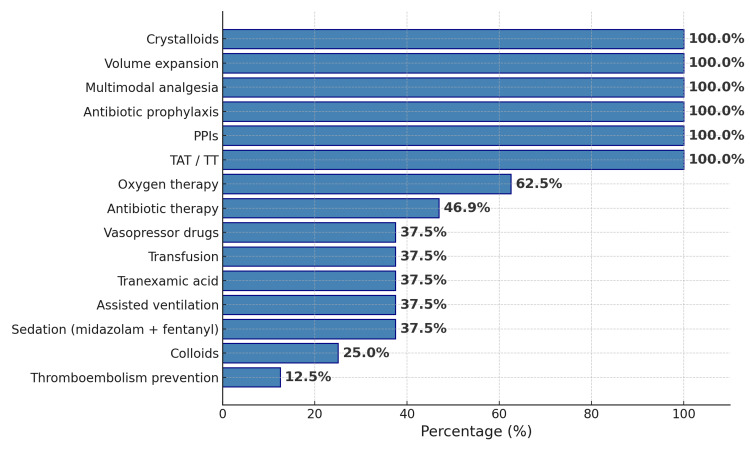
Combined distribution of treatments administered PPIs: Proton pump inhibitors; TAT/TT: Tetanus antitoxin/tetanus toxoid.

All patients received standardized initial management including crystalloid resuscitation, empiric antibiotics, proton pump inhibitors, tetanus prophylaxis, and multimodal analgesia. Oxygen therapy was administered in 62.5% (n = 20), and 37.5% (n = 12) required invasive mechanical ventilation. Central venous access was established in 12 cases. Vasopressor support and blood transfusion were each required in 37.5% of patients (n = 12); the mean transfusion volume was 22.5 units (SD ≈ 10.42). Colloid administration (Plasmion) was documented in 25% (n = 8), and pharmacologic thromboembolism prophylaxis was initiated in 12.5% (n = 4) (Figure 13).

Surgical outcomes

Of the 32 ICU-admitted patients, 87.5% (n = 28) underwent a single surgical procedure (SD ≈ 0.0586; 95% CI: 0.760-0.990), while 12.5% (n = 4) required multiple interventions, reflecting the complexity and recurrence of certain injury profiles (SD ≈ 5.85%; 95% CI: 1.04%-23.96%). Intensive care monitoring was required in 62.5% (n = 20), underscoring both the cohort’s critical status and the severity of associated physiological derangements. All patients received pharmacologic therapy, including fluid resuscitation, antibiotics, analgesia, and supportive care.

**Figure 14:**
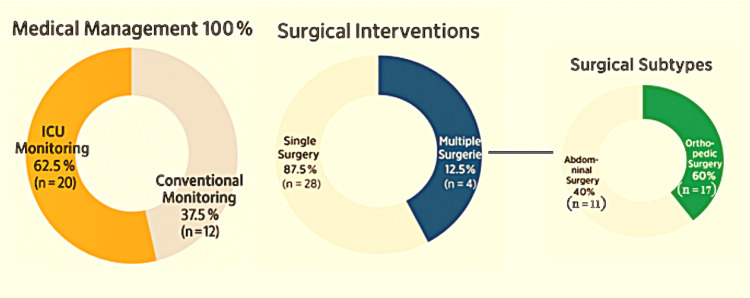
Composite pie showing the overall management strategy for ballistic trauma patients

Surgical subtypes were predominantly orthopedic (60%, n = 17), reflecting the high incidence of extremity trauma, followed by abdominal surgeries (40%, n = 11) addressing distension, gastrointestinal bleeding, penetrating wounds (each 37.5%, n = 12), and evisceration (12.5%, n = 4). Neurological impairment (GCS: 9-13) was observed in 37.5% (n = 12), and D12 spinal fractures with posterior wall displacement were present in 12.5% (n = 4). These findings emphasize the anatomically diverse and complex nature of ballistic trauma, with surgical strategies tailored to injury severity and location (Figure 14).

**Figure 15:**
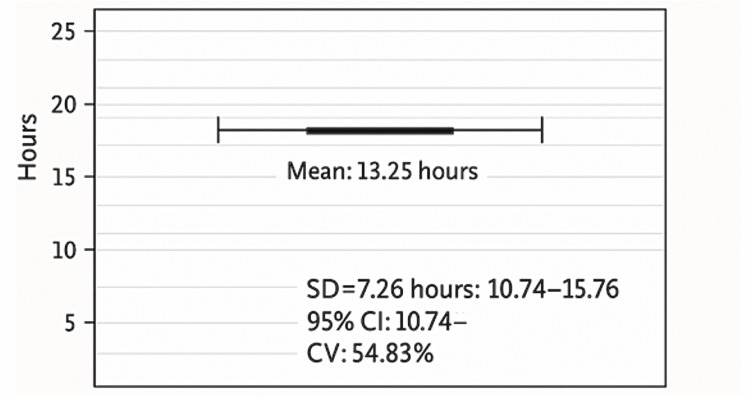
Time from trauma to surgery

The mean delay from trauma to operative intervention was 13.25 ± 7.26 hours (95% CI: 10.74-15.76; CV: 54.83%) (Figure 15).

Injury mechanism and severity

**Figure 16:**
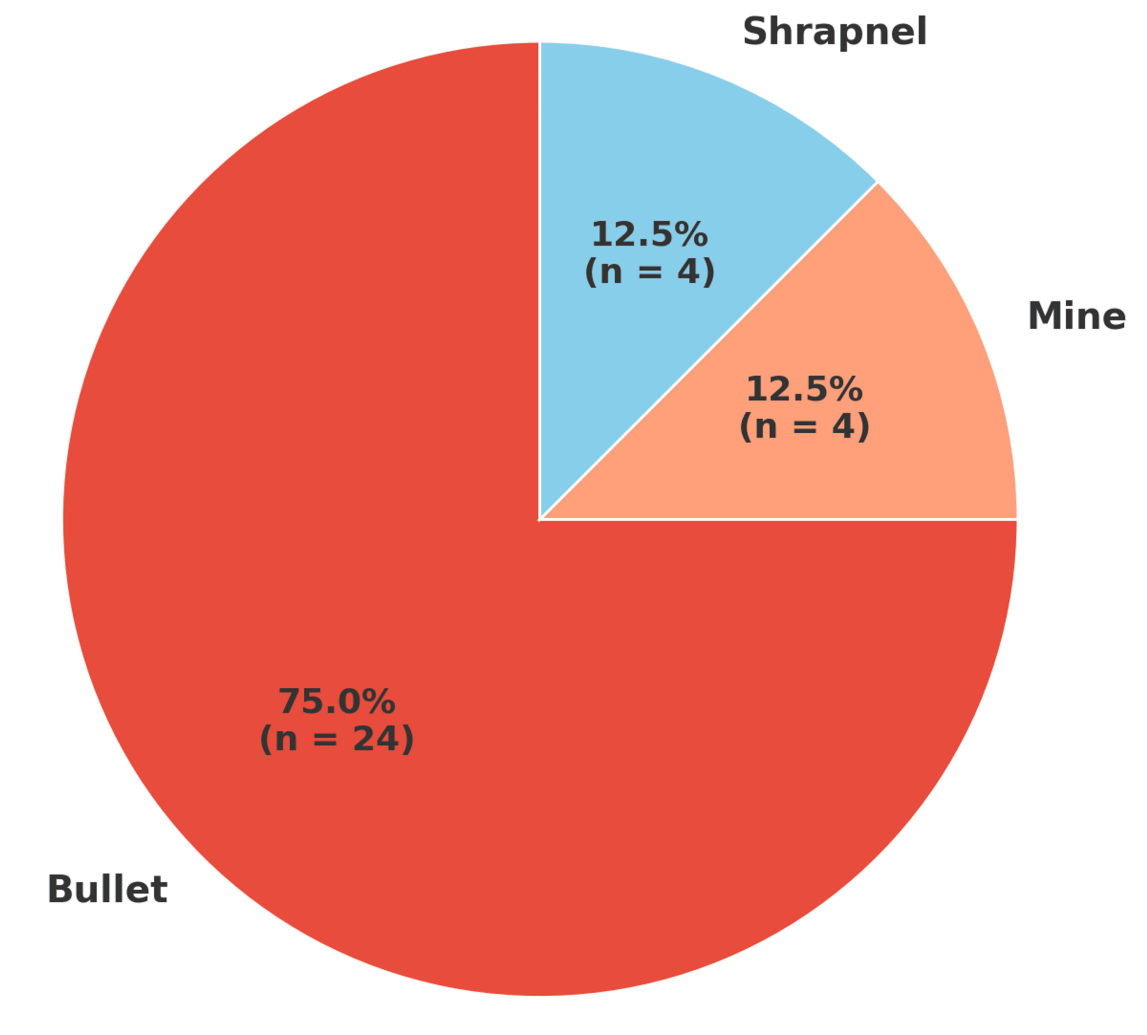
Distribution of patients according to the type of ammunition

Regarding trauma etiology, 75% (n = 24) of injuries resulted from bullets, while 12.5% (n = 4) were due to mines and 12.5% (n = 4) were due to shrapnel (Figure 16).

**Figure 17:**
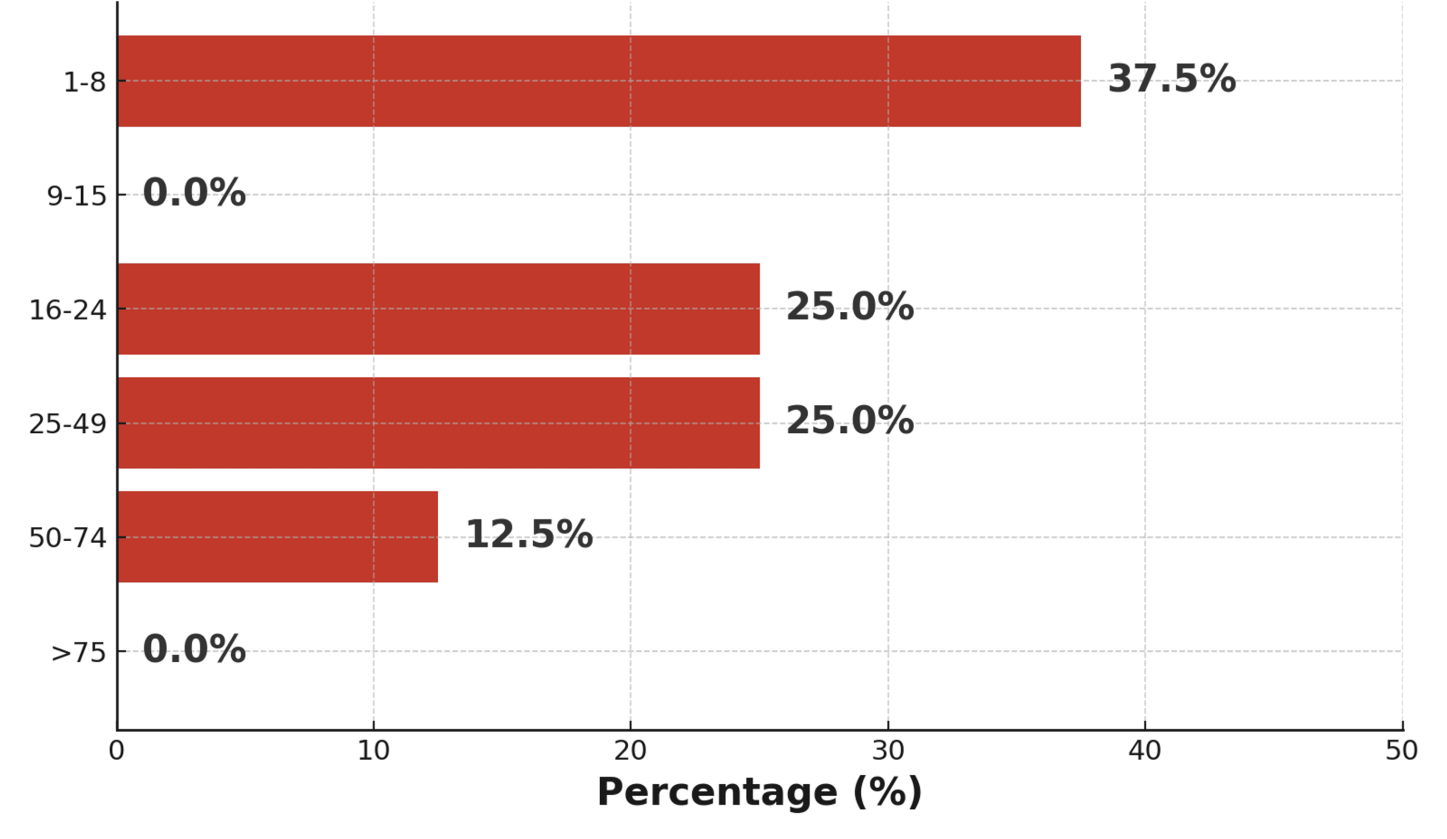
Distribution of patients according to Injury Severity Score (ISS)

Based on the Vittel triage criteria, 50% of patients (n = 16) were classified as having severe trauma. ISS ranged from 12 to 32 in 37.5% of cases (n = 12), indicating a predominance of moderate-to-severe injury profiles (Figure 17).

Prognostic indicators and clinical outcomes

**Figure 18:**
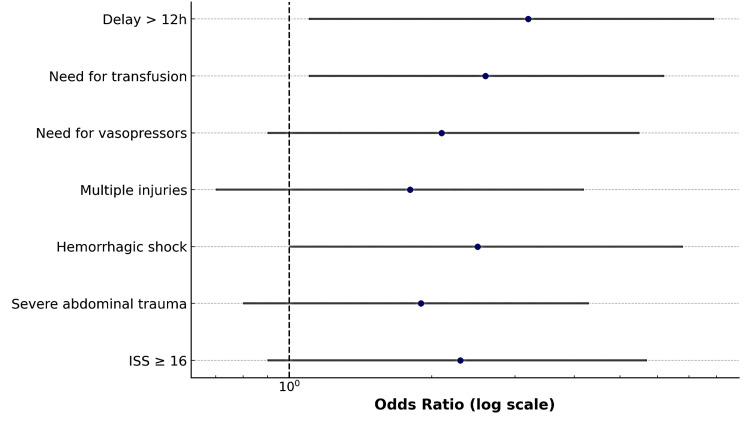
Forest plot, showing odds ratios with 95% confidence intervals for mortality predictors ISS: Injury Severity Score.

In this retrospective cohort of 32 ICU-admitted patients with ballistic trauma, adverse clinical outcomes, defined as mortality or major complications, were significantly associated with several early prognostic indicators. Notably, ISS ≥ 16, severe abdominal trauma, hemorrhagic shock, polytrauma, vasopressor use, transfusion requirement, and surgical delays exceeding 12 hours were all linked to increased risk, with ORs exceeding 1 despite wide CIs due to the limited sample size (Figure 18). Among these, the strongest associations were observed for transfusion need and delayed operative intervention.

Statistically significant predictors of unfavorable outcomes included older age (33.1 ± 6.2 vs. 29.5 ± 4.8 years; p < 0.05), hemodynamic instability upon ICU admission (p < 0.001), systolic hypotension (p = 0.02), MAP < 65 mmHg (p = 0.01), oliguria (p = 0.04), and the presence of comorbidities (41.7% vs. 20%; p = 0.03). Although tachypnea and tachycardia did not reach statistical significance (p = 0.08 and 0.09, respectively), their increased frequency suggested a trend toward greater physiological stress in non-survivors (Table 5). Due to the modest cohort size, multivariate logistic regression was not performed to avoid model overfitting; thus, all associations should be interpreted as exploratory.

**Table 5:** Comparative analysis of favorable vs. unfavorable outcomes SBP: Systolic blood pressure; MAP: Mean arterial pressure.

Characteristics	Favorable Outcomes (n = 20)	Unfavorable Outcomes (n = 12)	Test Statistic	P-value
Age (Mean ± SD)	29.5 ± 4.8	33.1 ± 6.2	t = 2.23	<0.05
Hemodynamic stability	100%	0%	χ² = 26.67	<0.001
Tachypnea	50%	83.3%	χ² = 2.98	0.08
Hypotension (SBP < 90 mmHg)	25%	66.7%	χ² = 5.38	0.02
MAP < 65 mmHg	15%	50%	χ² = 6.27	0.01
Tachycardia (HR > 100 bpm)	40%	66.7%	χ² = 2.84	0.09
Oliguria	15%	41.7%	χ² = 4.19	0.04
Comorbidity present	20%	41.7%	χ² = 4.42	0.03

Length of stay and mortality

**Figure 19:**
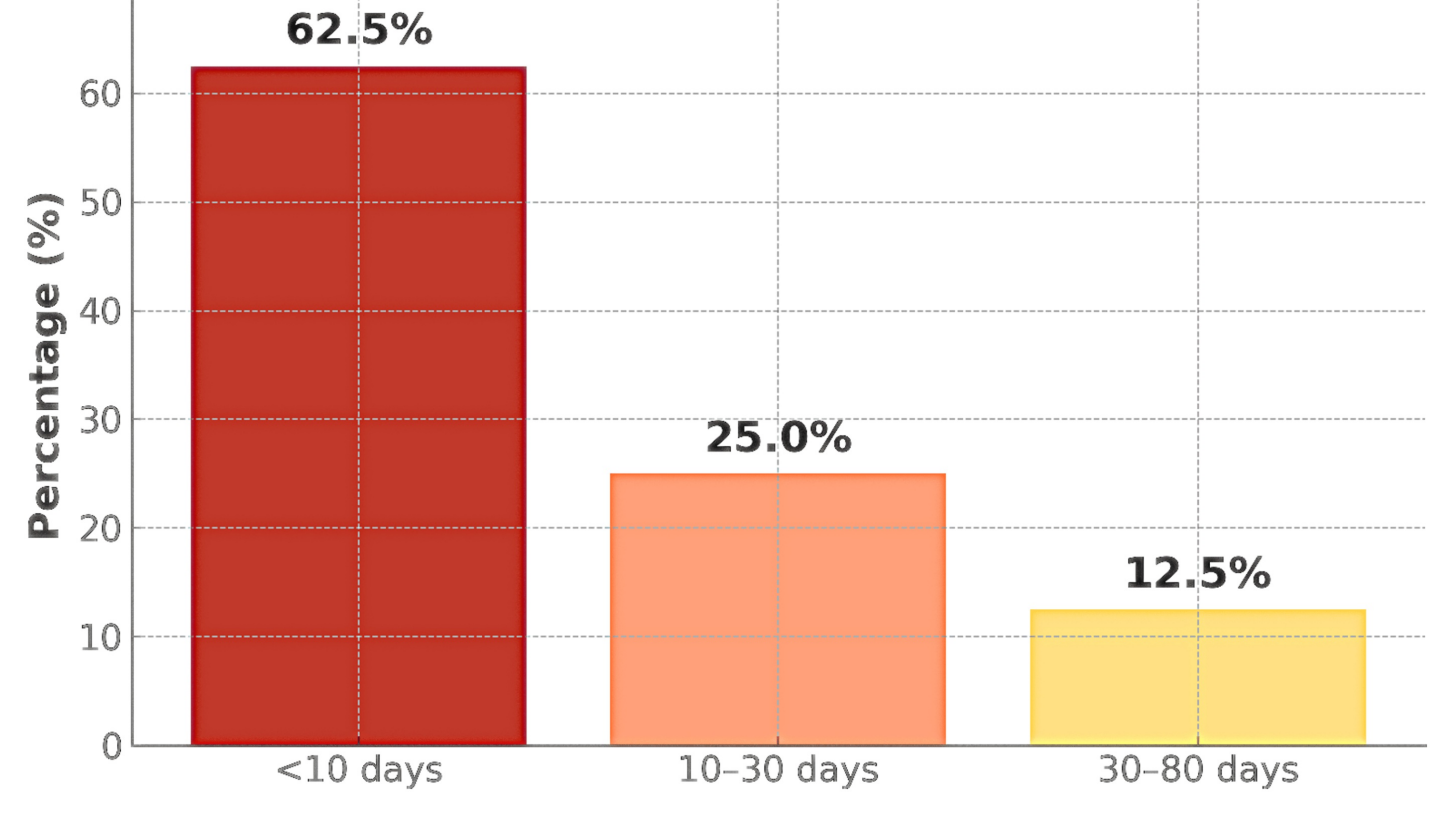
Distribution by length of hospital stay

Hospital length of stay was less than 10 days in the majority of patients 62.5% of cases (n = 20; standard deviation of the proportion ≈ 0.0856; 95% CI: 0.457-0.793) (Figure 19).

**Figure 20:**
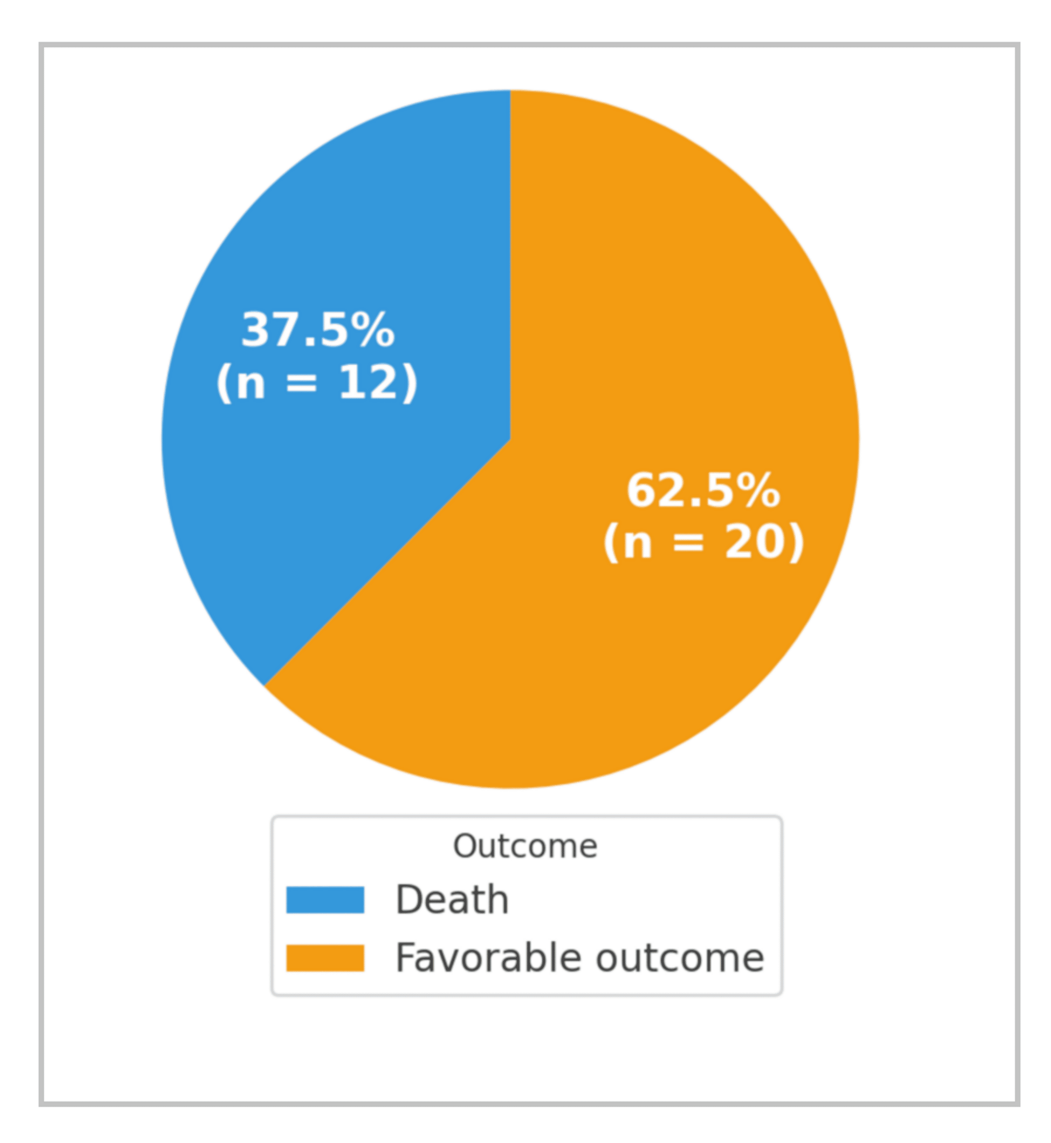
Distribution of patients according to outcomes

The overall mortality rate reached 37.5% (n = 12; 95% CI: 0.207-0.543). Favorable outcomes, defined as survival to discharge without major complications or permanent disability, were observed in 62.5% of patients (n = 20; SD ≈ 0.0856) (Figure 20). These findings, while reflective of established trauma outcome patterns, must be interpreted as exploratory due to the study’s retrospective design and limited sample size. Multivariate regression was not performed to avoid model overfitting.

## Discussion

This comprehensive six-year retrospective study offers valuable epidemiological and clinical insights into the intricate patterns, severity, and outcomes associated with ballistic trauma that necessitates intensive care management within the specific context of military training. The crucial findings from this research closely align with existing global literature that describes similar trauma patterns observed in conflict and combat simulation environments, particularly concerning key aspects such as patient demographics and the distribution of various types of injuries sustained. Such alignment underscores the importance of understanding these patterns to improve care strategies and outcomes for affected individuals in critical military training settings [1,4].

Limb trauma was the predominant injury in this cohort (50%), aligning with data from military trauma registries in conflicts such as Iraq and Afghanistan, where extremity injuries are frequently reported among ballistic cases [5]. These often-involved fractures require orthopedic intervention. Imaging modalities, notably radiography and CT (including angiographic studies), were essential for evaluating vascular injury and guiding surgical decisions, reinforcing current recommendations for early advanced imaging in trauma care [5,6]. Rather than emphasizing procedural variations, the findings illustrate the consistent use of established trauma principles, including timely stabilization, wound control, and functional preservation, based on institutional protocols aligned with international standards. These results highlight that structured, protocol-based approaches can yield effective outcomes in resource-limited military settings, even in the absence of advanced surgical innovations [7-12].

The management of complex abdominal injuries was primarily guided by CT-based imaging, including triple-contrast protocols for stable patients, and selective use of angiography for vascular assessment. In unstable cases, damage control strategies emphasizing rapid hemorrhage control and contamination limitation were prioritized. While life-saving procedures such as splenectomy and nephrectomy were undertaken, the study does not support detailed organ-specific prognostic conclusions. Instead, it highlights the broader systemic challenges of managing multisystem injuries in resource-limited, time-sensitive contexts [6,13-18].

In our cohort, the management of hollow viscus injuries focused on rapid control of contamination, particularly in unstable patients, where linear stapling was favored to reduce operative time, and stoma creation was generally avoided. One fatal case following stapled repair during advanced shock underscored the elevated risk of sepsis in delayed bowel injury management [15]. Standard midline laparotomy using cold blade dissection minimized intraoperative bleeding, and hemorrhage control was achieved through manual aortic compression and mesenteric root pressure, in line with established damage control protocols [19]. The prognosis was particularly poor in cases of complex multisystem abdominal trauma. A representative thoracoabdominal case with renal, mesocolic, and retroperitoneal involvement culminated in septic shock and death despite prompt surgical intervention, highlighting the high lethality of polytrauma compounded by multidrug-resistant infections [15].

Duodenal and pancreatic injuries, though infrequent in our cohort, underscore the diagnostic complexity of high-velocity abdominal trauma. While classical intraoperative indicators, such as bile staining and periduodenal hematoma, are well established, the retrospective design of our study did not permit procedural stratification or detailed outcome analysis. Pancreatic trauma was managed conservatively, with no cases requiring major resection beyond limited distal pancreatectomy, in line with contemporary trauma care guidelines [20,21]. Colonic and rectal injuries were managed according to standard trauma protocols, with surgical decisions based on hemodynamic stability and injury location. Strategies such as fecal diversion and presacral drainage were employed when clinically appropriate [22,23], though the dataset lacked sufficient granularity to assess long-term outcomes such as bowel function or continence preservation. Overall, while these operative approaches reflect adherence to internationally accepted trauma principles, the retrospective and ICU-focused nature of the study limits procedural specificity. The more meaningful insights lie in the systemic demands of managing multisystem ballistic trauma under time and resource constraints, emphasizing the critical value of early recognition, rapid triage, and structured trauma pathways over isolated surgical nuances.

Thoracic injuries in ballistic trauma presented complex diagnostic and therapeutic challenges, particularly due to subtle external signs. Following standard trauma protocols, imaging via eFAST, CT, and radiography was routinely employed in patients with precordial or transmediastinal injuries [24]. Sternal ballistic wounds proved universally fatal before ICU admission, while transdiaphragmatic injuries among survivors were associated with high morbidity, exemplified by a case complicated by colocutaneous fistula and mesocolonic infiltration [24,25].

Retroperitoneal hemorrhages (RPH) were classified as central, lateral, or pelvic. Central RPHs often involve major vascular injury requiring surgical control using established exposure techniques [18]. Pelvic bleeding was managed through preperitoneal pelvic packing in accordance with current damage control practices.

Renal trauma was graded per the American Association for the Surgery of Trauma (AAST) classification system. Conservative management was favored for hemodynamically stable Grade IV injuries, consistent with current nonoperative trends [26,27]. Grade V injuries necessitated surgical intervention in unstable patients, including nephrectomy and, in one case, concurrent right colectomy following CT angiography and laparotomy, with favorable outcomes. Select cases were managed with partial nephrectomy or angiographic embolization when feasible [27,28]. Temporary pedicle clamping was employed for rapid hemorrhage control in cases of exsanguination [29]. Due to limited case numbers, no definitive prognostic conclusions can be drawn, and procedural efficacy cannot be generalized.

In cases involving extensive visceral edema or requiring repeat exploration, temporary abdominal closure using sterile dressings and negative pressure wound therapy (NPWT) was utilized to reduce the risk of abdominal compartment syndrome (ACS), a serious complication linked to fluid overload and intra-abdominal trauma [29]. Early identification and decompression remain essential in trauma care.

In cases of suspected cardiac or great vessel injury, surgical strategy focused on hemorrhage control and stabilization. Unstable patients underwent left anterolateral thoracotomy for aortic access, while midline sternotomy was preferred in the absence of tamponade. Temporary vascular shunts and extracorporeal support were occasionally employed in complex scenarios following trauma guidelines [18]. Thoracic injuries were managed with chest drainage and, when necessary, surgical intervention, underscoring the role of early thoracic imaging.

Diaphragmatic injuries, identified via cross-sectional imaging, were addressed within the broader surgical strategy, with emphasis on timely intervention rather than anatomical classification [28]. Severe burns, though uncommon, were managed per international standards, including cooling, sterile dressing, infection prevention, and fluid resuscitation guided by the Rule of Nines [30].

Pelvic fractures were treated with early stabilization using trochanteric binders and preperitoneal packing, aligning with damage control orthopedics. Maxillofacial and cervical trauma required prompt airway protection and vascular control via the zone-based approach, while spinal injuries were managed through immobilization and decompression when feasible, with outcomes largely dependent on initial spinal cord damage [30].

These findings underscore the critical need for a coordinated trauma care system capable of providing standardized imaging, timely surgical intervention, and structured damage control strategies. In our context, early multidisciplinary decision-making and adherence to the principle of damage control at ground zero were key to achieving stabilization, despite logistical barriers and inconsistent prehospital timelines.

Consistent with military trauma registries from Iraq, Afghanistan, and Ukraine, our data confirm the predominance of extremity injuries, frequent thoracoabdominal trauma, and the routine use of damage control principles [6,9]. However, our cohort’s mortality rate of 37.5% exceeds the 10%-30% range reported in similar settings [4,9], likely reflecting delayed surgical access (mean delay: 13.25 ± 7.26 hours), underdeveloped prehospital systems, and the complexity of multisystem injuries, particularly involving thoracoabdominal and spinal regions.

Despite the challenges of military field operations, frontline teams applied essential life-saving measures, including tourniquet use, hemostatic dressings, airway positioning, needle decompression, and early vascular access [2,4]. These interventions, grounded in the damage control at ground zero concept, contributed to physiological stabilization during evacuation and laid the foundation for subsequent surgical and intensive care management.

The integration of these actions into structured triage protocols, combined with rapid transport and early use of antifibrinolytics such as TXA, is central to the "Golden Hour" doctrine and should be a priority in trauma system planning [4]. Standardized scoring tools, notably the ISS and Vittel triage criteria, proved valuable in identifying high-risk patients and guiding early clinical decisions, as 50% of our cohort met the threshold for severe trauma [9].

Management strategies followed international trauma surgery standards, focusing on early intervention, organ preservation, and staged surgical control. Nevertheless, outcomes were significantly impacted by injury severity, delayed presentation, and a high rate of septic complications. Physiological markers such as hypotension, MAP < 65 mmHg, and oliguria emerged as early indicators of poor prognosis, especially in patients with comorbidities or modest age increases in this otherwise young population.

While prehospital care was variably applied due to the constraints of military training settings, critical interventions, particularly hemorrhage control and airway management, were frequently initiated, supporting the value of field-based resuscitation. The observed surgical delay reflected systemic evacuation limitations rather than triage failures.

Importantly, the integration of advanced trauma care components, such as ultrasound-guided regional anesthesia, early transfusion strategies, and triple-contrast CT imaging, demonstrated the feasibility of aligning institutional practice with high-income trauma care standards, even in resource-limited environments [13,27].

Ultimately, our experience affirms that improved outcomes in high-energy ballistic trauma depend not on exhaustive procedural detail but on timely triage, early imaging, protocol-driven intervention, and multidisciplinary coordination. These principles, emphasized by NATO, Advanced Trauma Life Support (ATLS), and the American College of Surgeons, support the implementation of structured trauma systems that extend from prehospital stabilization to definitive surgical management [9,17,18]. Immediate field resuscitation remains the cornerstone of survival, particularly in mitigating hemorrhagic shock and enabling effective downstream care.

Nonetheless, several limitations must be acknowledged. The retrospective, single-center nature of the study inherently introduces risks of selection and information bias. Variability in the quality of clinical documentation, particularly in earlier records, may have affected the accuracy and completeness of critical variables, including physiological status at admission and prehospital interventions. Incomplete patient files, especially those lacking preoperative and postoperative imaging, limited the ability to assess radiological progression and fully characterize injury evolution. Although cases with missing essential data were excluded to preserve analytical validity, the absence of imputation may have further constrained robustness.

Due to the modest cohort size (n = 32), multivariate logistic regression was not performed to avoid model overfitting and unstable estimates. As a result, adjustment for key confounders such as age, comorbidities, and injury location was not feasible, and all reported associations should be interpreted as exploratory rather than confirmatory. Despite this limitation, the findings remain clinically informative and hypothesis-generating. They underscore the prognostic value of early physiological assessment and support the integration of individualized risk stratification into trauma triage algorithms.

Trauma severity scoring was based on retrospectively applied ISS and MESS classifications. However, interrater reliability testing was not conducted, and additional validated scoring tools such as the RTS and the Abbreviated Injury Scale (AIS) were not used. These methodological constraints may have limited the precision of injury stratification and reduced the external reproducibility of the findings. Future prospective studies incorporating larger, multicenter cohorts, standardized trauma registries, and adjusted analytical frameworks are needed to validate these preliminary associations and better define the independent prognostic contribution of each clinical determinant.

The absence of a comparator group (e.g., non-ICU trauma patients or matched civilian cases) further restricts external validity beyond the military ICU setting. Additionally, the lack of standardized follow-up precluded evaluation of long-term functional outcomes, disability, or quality of life. One case initially misclassified as ICU-admitted was excluded after a systematic eligibility review, reinforcing methodological rigor in response to peer critique.

These limitations underscore the urgent need for prospective, multicenter studies supported by standardized trauma registries, validated severity scoring systems, and structured long-term follow-up protocols. Incorporating prehospital data and ensuring the use of comprehensive injury stratification tools, such as ISS, RTS, and AIS, would facilitate more accurate risk adjustment and outcome modeling. Larger sample sizes across diverse institutions would enable robust multivariable analyses to identify independent predictors of mortality and adverse outcomes. Such studies are essential not only to strengthen the external validity and clinical applicability of prognostic indicators but also to improve the generalizability of findings across both military and civilian trauma systems exposed to high-energy ballistic injuries.

## Conclusions

This six-year retrospective cohort study underscores the severity and complexity of ballistic trauma sustained during military training, with a high mortality rate of 37.5% despite a predominantly young and healthy population. Extremity and abdominal injuries were the most common, while thoracic and spinal trauma presented significant challenges. Adverse outcomes were associated with hypotension, oliguria, comorbidities, and low MAP, indicating their potential as early prognostic markers. These findings highlight the importance of structured trauma scoring, early physiological assessment, damage control at ground zero, and timely surgical intervention, while also acknowledging the limitations inherent to the study’s retrospective, single-center design.
